# Muscle-driven simulations and experimental data of cycling

**DOI:** 10.1038/s41598-023-47945-5

**Published:** 2023-12-06

**Authors:** Caitlin E. Clancy, Anthony A. Gatti, Carmichael F. Ong, Monica R. Maly, Scott L. Delp

**Affiliations:** 1 Department of Mechanical Engineering, Stanford University, Stanford, CA USA; 2 Department of Radiology, Stanford University, Stanford, CA USA; 3 Department of Bioengineering, Stanford University, Stanford, CA USA; 4 Department of Kinesiology and Health Sciences, University of Waterloo, Waterloo, ON Canada; 5 Department of Orthopaedic Surgery, Stanford University, Stanford, CA USA

**Keywords:** Biomedical engineering, Scientific data

## Abstract

Muscle-driven simulations have provided valuable insights in studies of walking and running, but a set of freely available simulations and corresponding experimental data for cycling do not exist. The aim of this work was to develop a set of muscle-driven simulations of cycling and to validate them by comparison with experimental data. We used direct collocation to generate simulations of 16 participants cycling over a range of powers (40–216 W) and cadences (75–99 RPM) using two optimization objectives: a baseline objective that minimized muscle effort and a second objective that additionally minimized tibiofemoral joint forces. We tested the accuracy of the simulations by comparing the timing of active muscle forces in our baseline simulation to timing in experimental electromyography data. Adding a term in the objective function to minimize tibiofemoral forces preserved cycling power and kinematics, improved similarity between active muscle force timing and experimental electromyography, and decreased tibiofemoral joint reaction forces, which better matched previously reported in vivo measurements. The musculoskeletal models, muscle-driven simulations, simulation software, and experimental data are freely shared at https://simtk.org/projects/cycling_sim for others to reproduce these results and build upon this research.

## Introduction

More than 50 million Americans (12.4% of the population) cycle for sport, leisure, transportation, and rehabilitation^1^. Previous research has characterized cycling kinematics^2–4^, pedal forces^5–8^, joint moments^7–10^, and muscle activity with electromyography (EMG)^7,11–17^ by analyzing experimental data. Other work has focused on biomechanical consequences of altering power^3,5^, cadence^3,5,10,18,19^, and bike fit^2,20,21^. Previous studies encompass a wide range of cadence (40–120 RPM), power output (98–350 W), and cycling experience (recreational to elite).

Musculoskeletal simulation allows researchers to gain deeper insights into the biomechanics of movement. Simulation has been used to estimate biomechanical quantities that are hard to measure, such as muscle forces^22–24^, joint loads^25,26^, and muscle–tendon lengths and velocities^22,27^. Often, models are created and validated for specific applications, such as analyzing walking and running^28,29^, high-flexion human movements^14^, and knee contact forces^30^. Static optimization has been used to predict muscle forces in cycling^31^ and walking^32–34^, and to identify how alternate muscle coordination strategies may reduce knee contact forces^32,34^. Computed Muscle Control has been used to predict muscle excitations during cycling^14,35^, but unlike static optimization, Computed Muscle Control accounts for muscle–tendon dynamics, improving estimates of muscle fiber lengths and velocities; these muscle parameters are necessary for accurately estimating quantities such as the metabolic energy expended by muscles^36,37^. Computed Muscle Control, however, has limited flexibility in the optimization objective function, which only minimizes the sum of squared muscle activations.

Choosing an objective function when generating a simulation is important as the objective function represents the balance between many factors that affect movement. Adjusting the objective function enables simulations to be used to explore how a desired mechanical or physiologic outcome can be achieved or to test neuromuscular control hypotheses^38^. For example, simulations suggested that tibiofemoral reaction forces during gait can be reduced by more than 1 body weight, a desirable outcome for knee osteoarthritis patients, via changes in muscle coordination^34,39^. A follow-up experimental study showed that these changes in muscle coordination can be achieved using EMG biofeedback^32^. Furthermore, it has been suggested via an animal model^40^ and human experimental studies^41,42^ that neuromuscular coordination may be regulated by joint stresses and pain. Therefore, an objective function that captures key features for how humans control movement may need to include a term that represents joint reaction forces.

Recent advances and open-source tools in optimal control methods, and in particular direct collocation, allow researchers to efficiently solve trajectory optimization problems with many objectives and constraints^43^. These tools enable optimization of multiple concurrent objectives, such as muscle excitations, metabolic cost, and joint forces, which allows simulations to identify ways to improve human performance, optimize rehabilitation, or mitigate injury^43–46^. Important early work demonstrated that optimal control methods can successfully solve cycling-based trajectory optimization problems using planar models with torque actuators^47^ and with up to 9 muscles per leg^15,48^. Development of three-dimensional (3D) models will enable assessment of frontal plane loads during cycling, a particular interest for knee osteoarthritis rehabilitation^49–54^.

Freely available musculoskeletal models, software, and data provided by the Full-Body Gait Model^28^ and Full Body Running Model^29^ have enabled significant progress in biomechanics research for walking and running. Analogous freely available models, software, and data are not available for cycling that is representative of typical cadence and power. Thus, our primary aim was to develop a set of 3D muscle-driven simulations of cycling that capture the salient features of cycling biomechanics under a breadth of recreational conditions. The secondary aim was to explore how adjustments to the optimal control objective function could be used to identify muscle coordination patterns that reduce knee joint reaction forces during cycling. To enable other researchers to reproduce and build upon our work, we provide OpenSim models, marker trajectory data, pedal reaction forces, and code to run the direct collocation simulations at https://simtk.org/projects/cycling_sim.

## Methods

**Figure 1:**
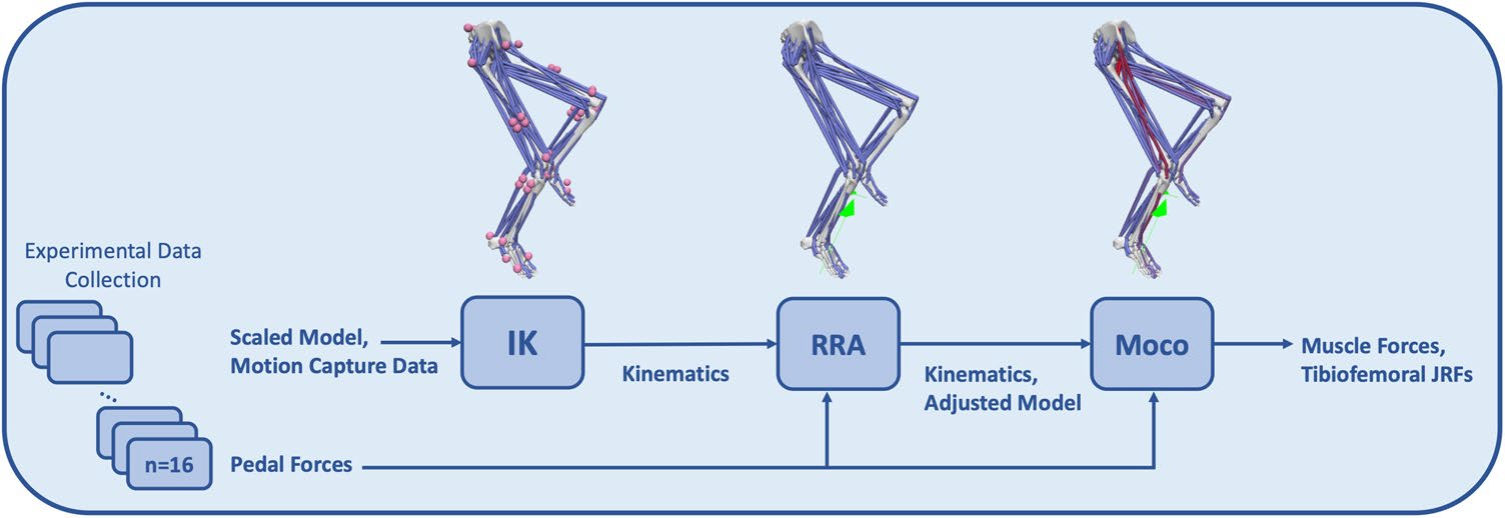
Motion capture and pedal force data were collected, and a musculoskeletal model was scaled for each participant. For each participant, the motion capture data and scaled model were used to perform inverse kinematics (IK). The pedal forces and resultant kinematics were input into the residual reduction algorithm (RRA) to produce dynamically consistent kinematics with a correspondingly adjusted model. These outputs, along with the pedal forces, were used in OpenSim Moco to generate muscle-driven simulations and calculate muscle forces and tibiofemoral joint reaction forces (JRF)s.

OpenSim^55^ was used to create 3D muscle-driven cycling simulations of healthy participants (Fig. 1). Scaled OpenSim models and experimental marker data^31^ served as inputs to the Inverse Kinematics (IK) Tool for computing joint kinematics. Errors in dynamic consistency between the kinematic and kinetic data were reduced using the Residual Reduction Algorithm (RRA) Tool. The OpenSim Moco^43^ Tool was then used to generate muscle-driven simulations to estimate muscle forces and tibiofemoral joint reaction forces.

Experimental data were collected in a previous study for 16 healthy participants (18–45 years of age) with cycling experience ranging from recreational to elite^21,31^. Participants provided informed consent, and this study was approved by the Hamilton Integrated Research Ethics Board and was carried out in accordance with all pertinent guidelines and regulations. Anthropometric data including height, inseam, and foot length were measured and used to prescribe a bike fit for each participant. All cycling was performed on a commercial bike (Fit Bike Pro, Purely Custom, Twin Falls, ID, USA) using flat, instrumented pedals with the foot tightly secured using Velcro straps. The participants cycled at a self-selected cadence and a power output (Table 1) that elicited a heart rate of 70–75% of age-predicted maximum^21,31^. Data were collected for three minutes of seated cycling. To track kinematics, 40 retroreflective markers were sampled at 112.5 Hz with 12 infrared cameras (Motion Analysis Corporation, Santa Rosa, CA). Synchronous pedal reaction forces were collected at 450 Hz (Science To Practice, Ljubljana, Slovenia). Four markers were placed on each pedal to track the pedals’ location and orientation. Marker data were filtered with a second-order low-pass dual-pass Butterworth 6 Hz filter (Python Software Foundation, python.org; SciPy, Enthought, SciPy.org) and pedal reaction force data were filtered with a low-pass 10 Hz filter (MATLAB R2020b, The MathWorks, Inc., Natick, MA, USA).

**Table 1:** Descriptive statistics of the included participants and their cycling trials.

Participant	Height (cm)	Weight (kg)	Sex	Cadence (RPM)	Power (W)	Age (years)
P01	169.0	60.4	Female	77	60	29
P02	182.2	92.3	Male	80	107	22
P03	171.2	74.2	Male	83	120	21
P04	166.2	61.1	Female	92	81	23
P05	195.4	90.8	Male	84	136	28
P06	181.7	77.3	Male	90	216	27
P07	188.1	79.4	Male	92	161	41
P08	173.0	66.5	Male	90	117	42
P09	179.8	76.1	Male	75	156	26
P10	183.5	89.3	Male	85	160	19
P11	163.1	54.2	Female	78	94	32
P12	183.7	91.5	Male	80	116	31
P13	191.6	83.0	Male	90	199	44
P14	159.8	56.7	Female	86	40	42
P15	168.5	57.1	Female	85	144	31
P16	179.6	78.0	Male	89	208	35

Participant-specific, 3D, 16 degree of freedom (DOF) lower-body musculoskeletal models (6 pelvis DOF, 3 hip DOF, 1 knee DOF, 1 ankle DOF) were developed for cycling based on a previous high flexion model^14^ (Supplementary Discussion S1). From the cycling trials, functional knee joint centers were created using the Score method^56^, and hip joint centers were calculated using the Harrington method^57^. Using joint centers and anatomic markers, the model was scaled for each participant using the OpenSim Scale Tool^21,31^. After scaling, muscle moment arms were verified manually, with particular emphasis on the biceps femoris which has been reported to erroneously produce knee extension moments in deep knee flexion^14^ (Supplementary Fig. 1).

The model was adjusted for use with the direct collocation method as implemented by OpenSim Moco^43^. Muscles were modeled using a continuous and differentiable muscle model^58^. For each muscle–tendon unit whose tendon slack length was shorter than its optimal fiber length, the tendon was modeled as rigid^14,28^. To model the pelvis-saddle interaction, actuators were applied to all 6 pelvis DOFs with sufficient capacity to support up to 1 body weight. All rotational degrees of freedom had torque actuators to ensure the model could produce the prescribed motions. With the exception of the torque actuator supporting hip rotation, in which the model was deemed to have insufficient muscle actuation^59^, all other torque actuators contributed joint moments that were within the acceptable range of 5% of the peak net moment for each respective degree of freedom^60^.

The OpenSim IK, RRA, and Moco simulation pipeline (Fig. 1) was analyzed for one full crank cycle of the right leg, two minutes into the cycling bout. A revolution of the crank cycle began when the right foot was at top dead center (TDC) and concluded once the right foot was back at TDC. The IK Tool minimized the least squares difference between motion capture markers and model markers to produce model kinematics. The RRA Tool filtered the IK kinematics at 6 Hz, and then used the filtered kinematics and filtered pedal reaction forces and moments applied to the calcaneus to adjust the kinematics and model’s segment masses and mass center locations to improve dynamic consistency^61^. Finally, OpenSim Moco was used to perform direct collocation to generate muscle-driven simulations and to compute the muscle forces and tibiofemoral forces that produced each cycling trial’s kinematics^43^.

The MocoInverse Tool was used to solve for muscle excitations necessary for the RRA-adjusted model to match the prescribed RRA-adjusted kinematics and pedal reaction forces. To compute excitations, the optimizer minimized an objective function subject to kinematic constraints^43^. MocoInverse enforces muscle excitations and muscle activations to be equal at the start of the simulation. Simulations were started before TDC, and then only the data from a single revolution (TDC to TDC) were analyzed. Two different optimization functions were implemented. The first objective function (*J*_*1*_) was used to generate baseline simulations that produced the desired cycling motion while minimizing muscle effort. The optimizer sought to minimize *J*_*1*_, which was composed of the weighted sum of a control effort term (*w*_*1*_ = 2) and an implicit auxiliary derivatives term (*w*_*2*_ = 1e−6). The control effort term included both the sum of squared muscle excitations (*e*(*t*)) and the sum of squared reserve and residual actuator controls at each of the DOFs (*u*(*t*)) from the start (*t*_0_) to the end (*t*_f_) of the simulation. In our model, muscle excitations (*e*(*t*)) are control inputs that range between 0 and 1, representing neural excitation (motor neuron recruitment and firing rate), and forces are calculated from excitations using a muscle model^62^. Residual actuators account for unmeasured saddle-pelvis interactions. Reserve actuators supply additional torque for lower-body degrees of freedom, which can help improve convergence^60^. The control effort weight (*w*_*1*_ = 2) was chosen by testing progressively smaller weights to reduce solver time while remaining sufficiently high enough that muscle activations were not sensitive to the exact weight chosen^43^. The implicit auxiliary derivatives term was defined as the sum of squared derivatives of the auxiliary variables related to compliant tendons ($\dot{z}$(*t*)), which improved convergence time. The auxiliary derivatives weight (*w*_*2*_ = 1e−6) was chosen such that it was sufficiently small to not substantially alter muscle excitations. The second objective function (*J*_*2*_) was used to study how muscle coordination can change in order to minimize knee forces. The second objective function augmented the first by adding a weighted (*w*_*3*_ = 1e−3) term to minimize the sum of squared tibiofemoral forces (*F*_tf_) of both legs. The optimizer convergence tolerance and optimizer constraint tolerance for all simulations were set to 1e−3. For clarity, we report all results for the right leg only.

1 $$J_{1} = w_{1} \left( {\mathop \sum \limits_{i = 1}^{{n_{{{\text{muscles}}}} }} \mathop \smallint \limits_{{t_{0} }}^{{t_{{\text{f}}} }} e_{i}^{2} \left( t \right)dt + \mathop \sum \limits_{j = 1}^{{n_{{{\text{actuators}}}} }} \mathop \smallint \limits_{{t_{0} }}^{{t_{{\text{f}}} }} u_{j}^{2} \left( t \right)dt} \right) + w_{2} \left( {\mathop \sum \limits_{k = 1}^{{n_{z} }} \mathop \smallint \limits_{{t_{0} }}^{{t_{{\text{f}}} }} \dot{z}_{k}^{2} \left( t \right)dt} \right)$$

2 $$J_{2} = J_{1} + w_{3} \left( {\mathop \sum \limits_{i = 1}^{{n_{{{\text{legs}}}} }} \mathop \smallint \limits_{{t_{0} }}^{{t_{{\text{f}}} }} {F_{{{\text{tf}}}} } _{i}^{2}\left( t\right) dt} \right)$$

## Results

**Figure 2:**
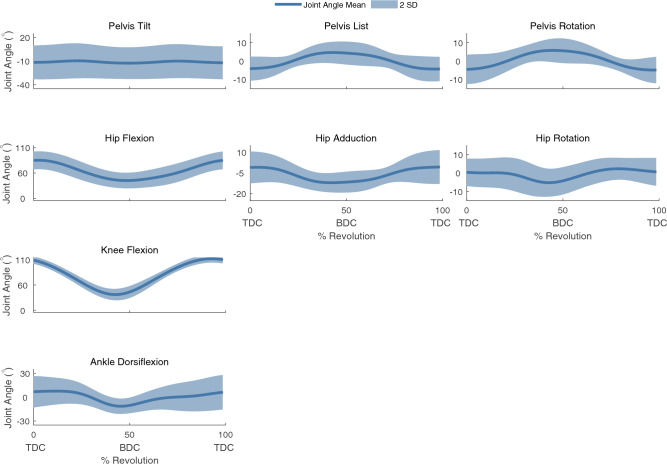
Simulation results of the mean joint angle ± 2 SD of the right leg for each rotational DOF. TDC denotes top dead center and BDC denotes bottom dead center of the crank cycle. Positive angles denote hip flexion, hip adduction, hip internal rotation, knee flexion, and ankle dorsiflexion. Pelvis tilt, list, and rotation are defined as rotation about the medial–lateral, anterior–posterior, and superior-inferior axes of the pelvis, respectively, and the positive directions of the axes of rotation for tilt, list, and rotation, are right, anterior, and superior in the pelvis reference frame.

**Figure 3:**
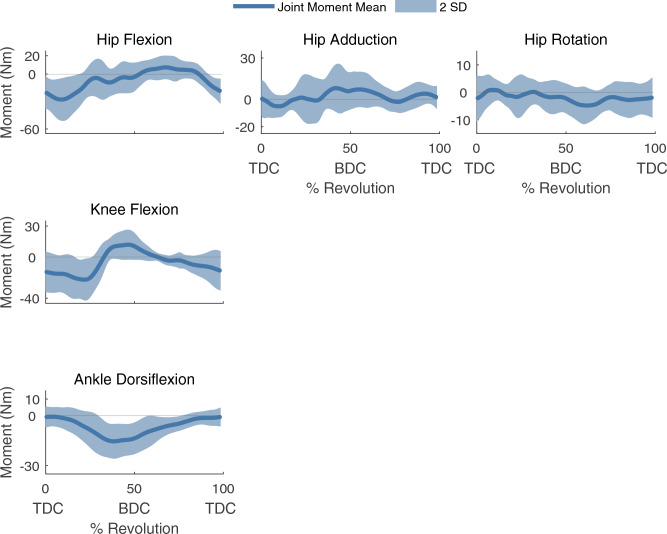
The mean joint moment ± 2 SD of the right leg for each rotational DOF. TDC denotes top dead center and BDC denotes bottom dead center of the crank cycle. Positive angles denote hip flexion, hip adduction, hip internal rotation, knee flexion, and ankle dorsiflexion. Hip flexion, knee flexion, and ankle dorsiflexion had relatively large inter-subject variability, likely due to differences in cycling power.

We observed that ankle dorsiflexion and pelvic tilt angles had high inter-subject variability, while knee and hip flexion had less (Fig. 2, Supplementary Fig. 2). Hip flexion, knee flexion, and ankle dorsiflexion joint moments also had relatively large inter-subject variability (Fig. 3, Table 1). Individual curves for all 16 participants for joint angles and joint moments are provided in Supplementary Figs. 2, 3. The kinematics of the simulation and those produced by IK were nearly identical (Supplementary Fig. 4).

**Figure 4:**
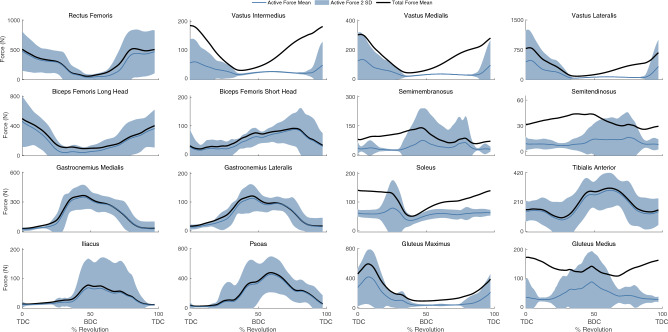
The muscle forces for 16 muscle–tendon units of the right leg with the baseline objective function (*J*_*1*_). The mean active muscle force for each muscle ± 2 SD is plotted in blue, while the total (passive + active) muscle force for each muscle is plotted in black. Please note, the y-axes are not constant between subplots.

**Figure 5:**
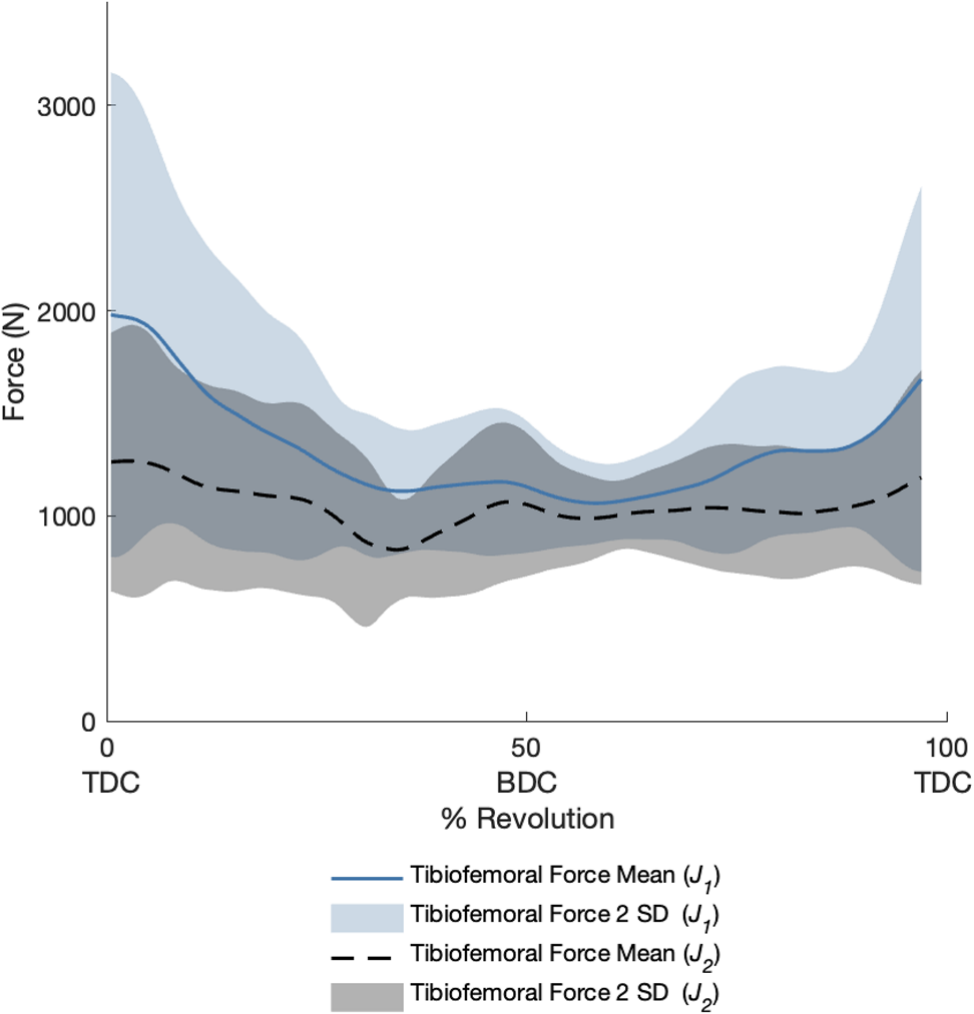
The mean compressive tibiofemoral forces ± 2 SD with an objective cost function, *J*_*1*_, minimizing muscle excitations only (solid line, light shading) and an objective cost function, *J*_*2*_, minimizing muscle excitations and tibiofemoral forces (dashed line, dark shading). Data for the right leg are presented. With the addition of a tibiofemoral force penalty (*J*_*2*_), tibiofemoral forces were reduced throughout the revolution.

For our baseline simulations (*J*_*1*_) that minimized muscle effort, the active muscle forces required to actuate the model had relatively large inter-subject differences for the muscles producing large active forces such as the rectus femoris, biceps femoris long head, tibialis anterior, iliacus, and psoas muscles, and smaller inter-subject differences for muscles producing smaller active forces such as the biceps femoris short head, semitendinosus, semimembranosus, and soleus (Fig. 4). Within-subject, between pedal revolution, variations in muscle forces were relatively small (Supplementary Fig. 5), thus aggregated muscle force data across all subjects are reported using a single revolution per subject. Muscle activations are also included in Supplementary Fig. 6. Passive forces, based on model kinematics and muscle passive force–length curves, contributed considerably to the total forces in the vasti, semimembranosus, semitendinosus, soleus, and glutei. Calculated tibiofemoral forces produced the characteristic first and second peaks, which occur just after TDC and just before bottom dead center (BDC) (Fig. 5).

**Figure 6:**
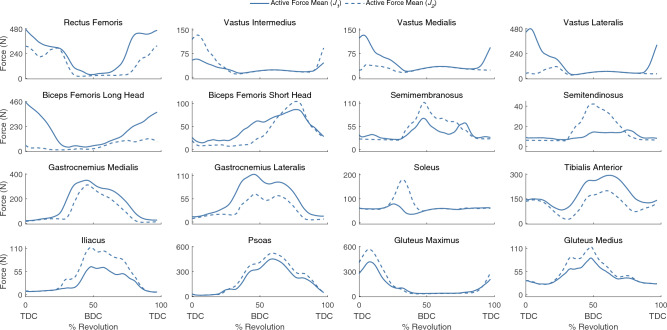
The mean active muscle forces for 16 muscle–tendon units of the right leg in the baseline simulations (*J*_*1*_, solid line) and simulations with an additional objective cost function minimizing tibiofemoral force (*J*_*2*_, dashed line). With the addition of a tibiofemoral force penalty, the gastrocnemii and biceps femoris long head active forces decreased; the soleus, semimembranosus, and semitendinosus active forces increased; and all quadriceps active forces decreased except for the vastus intermedius. Please note, the y-axes are not constant between subplots.

**Figure 7:**
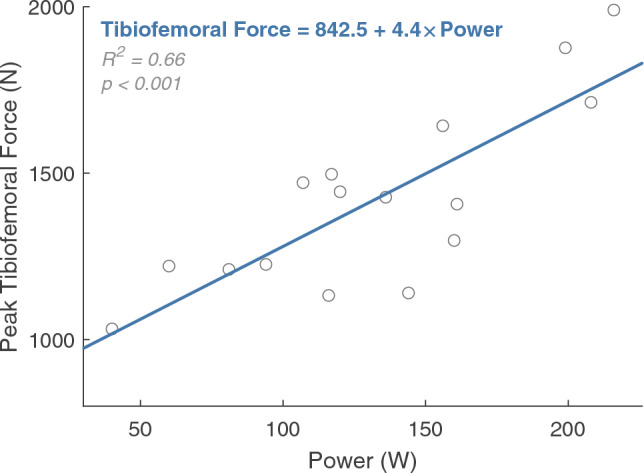
Relationship between each participant’s cycling power and the peak tibiofemoral force calculated from simulations run with a tibiofemoral force penalty in the objective function (*J*_*2*_). Included in the figure is the fitted simple linear regression model for predicting tibiofemoral force (N) from cycling power (W).

When tibiofemoral forces were also minimized in the objective function (*J*_*2*_), co-contraction of muscles that cross the knee was reduced (Fig. 6; Supplementary Fig. 6; Supplementary Fig. 7). For example, the gastrocnemii may be preferentially chosen over the soleus when the tibiofemoral force is not penalized (*J*_*1*_) because the model’s gastrocnemii have greater moment arms than the soleus moment arm over the course of a pedal revolution (Supplementary Fig. 8). The gastrocnemii are also active through BDC, when the knee has a net flexor moment and the ankle a plantarflexor moment; therefore, the gastrocnemii may serve dual purposes of generating plantarflexion and knee flexion moments. Active force was decreased in other major muscles that cross the knee, including the biceps femoris long head, biceps femoris short head, rectus femoris, and vastus lateralis; these changes in muscle forces decreased the mean tibiofemoral force for the entire crank cycle (Fig. 5). For simulations with and without the tibiofemoral force penalty, the primary tibiofemoral force peak occurred shortly after TDC. In simulations that were generated using the *J*_*2*_ objective function, a participant’s cycling power was a strong predictor of peak tibiofemoral forces (Fig. 7).

## Discussion

We developed muscle-driven cycling simulations using direct collocation for 16 participants. The participants spanned large ranges of cadence (75–99 RPM) and power (40–216 W), representative of experimental literature. We showed an example of how these data and models could be used to inform rehabilitation strategies by altering the objective function to produce muscle coordination strategies that reduce knee forces. The simulations identified that tibiofemoral forces can be reduced by increasing soleus force and reducing gastrocnemii forces, something that previous research indicates is trainable in a single session^32^.

Our direct collocation-based 3D simulations of cycling build upon previous work. Zignoli and colleagues created a 2D, torque-driven model for one participant and demonstrated that their model and optimal control framework could predict experimental joint torques that qualitatively compared well with experimental data of cycling^47^. Park et al.^15^ developed direct collocation-based cycling simulations with planar (2D) models driven by 9 muscle–tendon units per leg representing the major muscle groups that drive sagittal plane motion. Their two-legged model was first used to perform sensitivity analyses to investigate how different objective function weights, node densities, and initial conditions affect the results and time to solve the direct collocation problem. They then used a single-legged model^48^ to study how muscle coordination changed before and after learning a new way to direct pedal force. Park and colleagues freely shared their model and code^15^, a significant contribution to the field. However, the relatively low cadence and power (30 RPM and 30 W), even compared to older-adult clinical populations (40–90 RPM and 25–100 W)^51,63–66^, means these data have limited generalizability. Furthermore, the sagittal-plane nature of these models necessitates omission of frontal plane forces. Our work contributes 16, 3D participant-specific bi-lateral simulations driven by 80 muscle–tendon units, allowing for investigation of muscle actions and analysis of frontal plane biomechanics^49,50,52–54^.

With the tibiofemoral force penalty, tibiofemoral forces and muscle forces more closely matched experimental results^63^. Specifically, the mean peak tibiofemoral force was reduced from ~ 2000 to ~ 1200 N. These reduced forces better agree with in vivo measurements from an instrumented joint replacement where peak forces were between 793 and 1520 N when cycling at 120 W^63^ (mean power of our study = 137 W). Furthermore, our model’s estimated tibiofemoral forces were related to cycling power (Fig. 7; *R*^*2*^ = 0.66, *p* < 0.001), agreeing with results from the instrumented joint replacements^63^. Muscle force changes included a decrease in biceps femoris long head muscle forces near TDC, and an increase in semimembranosus and semitendinosus muscle forces near BDC; both changes better agree with EMG data from the literature^7,11–17^. Furthermore, peak soleus muscle forces increased substantially, while the gastrocnemii and tibialis anterior muscle forces decreased. The resulting plantarflexor muscle force patterns were more consistent with EMG data in the literature, particularly the increased soleus activity^7,12–17^. Therefore, for cycling, tibiofemoral force penalization may create a more physiologic simulation due to improved force sharing between ankle plantarflexors^7,11–17^. Future research should continue to explore whether inclusion of a tibiofemoral force penalty term in the objective function better represents human motor control patterns^41^.

The model’s simulated muscle forces capture salient features present in previous EMG literature. However, there were a few discrepancies. In particular, the biceps femoris long head was not active at BDC, as seen in the literature, and there was delayed active force timing of the biceps femoris short head^7,11–16^. Delayed timing may be explained by electromechanical delay between EMG data and force production^67^. It is possible that excess vasti passive forces, which comprised a significant portion of their total force, could have affected timing and magnitudes of the active hamstring forces (Supplementary Fig. 9). Considerable passive forces, relative to total forces, also existed for the soleus, semimembranosus, and glutei (Supplementary Fig. 9). After adjusting the soleus optimal fiber lengths (Supplementary Fig. 10; Supplementary Discussion S1), mean passive forces in the model were less than 8% of their respective maximum isometric forces, and thus were deemed acceptable. Unfortunately, we did not have experimental EMG data for direct comparison with our trials.

We present a set of baseline cycling simulations and an example set of simulations generated by penalizing tibiofemoral forces that resulted in an alternative, potentially more physiologic, muscle coordination strategy. We encourage researchers to build upon this work by leveraging the accompanying data and the flexibility of the direct collocation method. For instance, alternate cost terms can be added to these simulations to identify optimal muscle control strategies for different clinical or human performance applications. Due to the adaptability of the bicycle, another avenue for future research includes using muscle-driven predictive simulations (e.g., those that allow joint kinematics to deviate from experimental data) to gain insight into optimizing joint kinematics, muscle coordination, and bike fit. These simulations could help identify bike fits that reduce injury risk through, for example, minimizing peak patellofemoral joint reaction forces for patellofemoral pain or Achilles forces for Achilles tendinopathy. The use of muscle-driven simulations applied to cycling has the potential to have a high impact for optimizing rehabilitation and human performance.

### Supplementary Information

Supplementary Information.

## Data Availability

All data and code to run the presented simulations are freely available at https://simtk.org/projects/cycling_sim.
