# Supplementary material for: Muscle-driven simulations and experimental data of cycling
Source: Sci Rep. 2023 Dec 6;13:21534. doi: 10.1038/s41598-023-47945-5 (PMC10700567; doi:10.1038/s41598-023-47945-5)
Supplement: Supplementary file 1 — Supplementary Information. [file 41598_2023_47945_MOESM1_ESM.pdf]

**Supplementary**

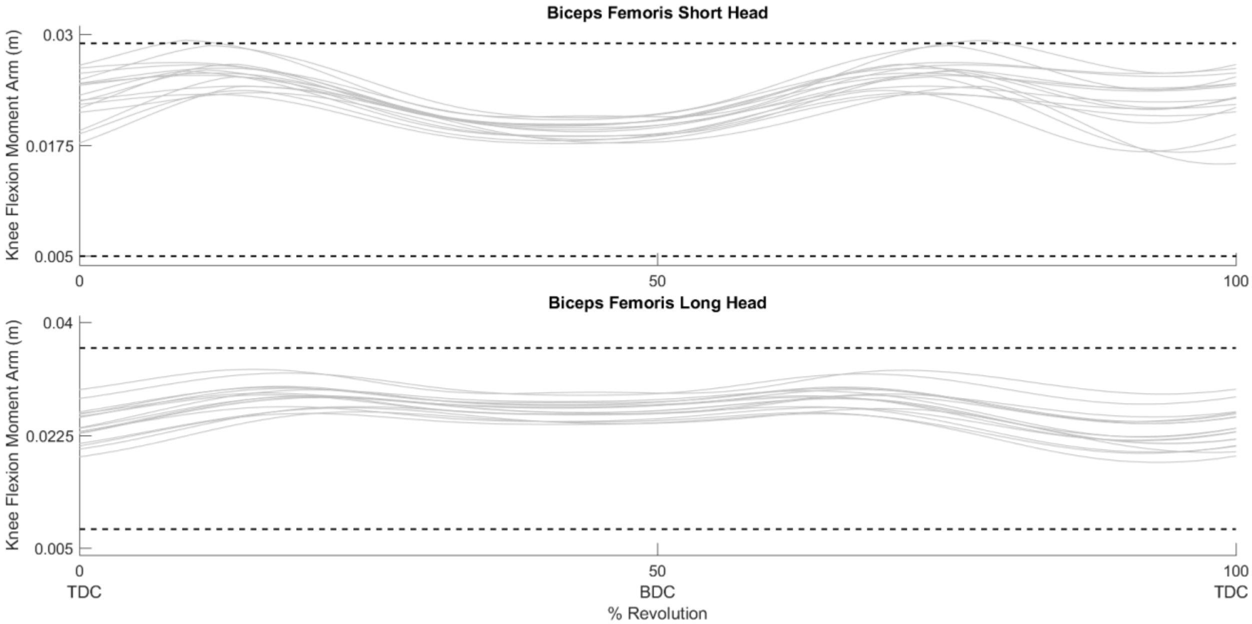


**Supplementary Figure 1.** Biceps femoris short head and long head moment arms over a pedal revolution for all subjects. Dashed lines show the range of moment arms for each respective muscle observed between ~5-130 degrees of knee flexion, as measured from cadaveric data. All but one participant lies within this experimental data range, indicating the biceps femoris moment arms are within the physiologic range. The cadaveric moment arm range was determined by taking the minimum and maximum values of the mean +/- 1 standard deviation moment arm vs. knee flexion angle curves from Buford et al. 1997 Figure 8.

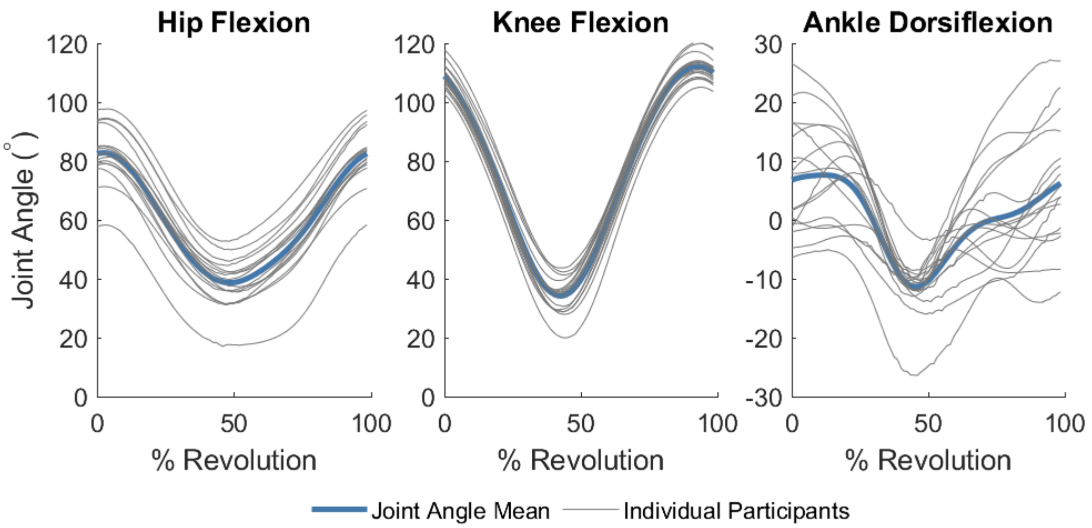


**Supplementary Figure 2.** Joint angles of each individual participant (gray) and the mean of the group (blue), n=16.

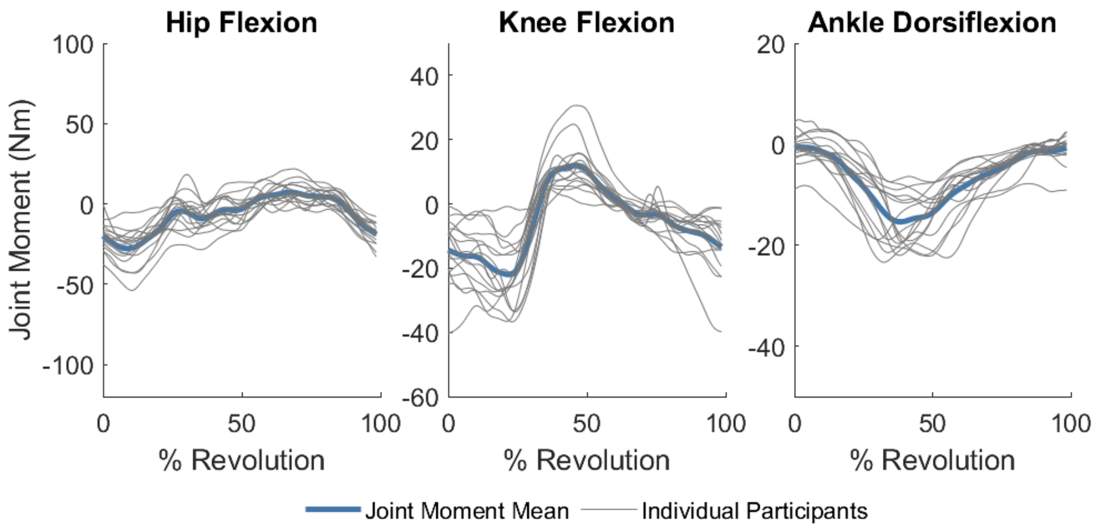


**Supplementary Figure 3.** Joint moments of each individual participant (gray) and the mean of the group (blue), n=16.

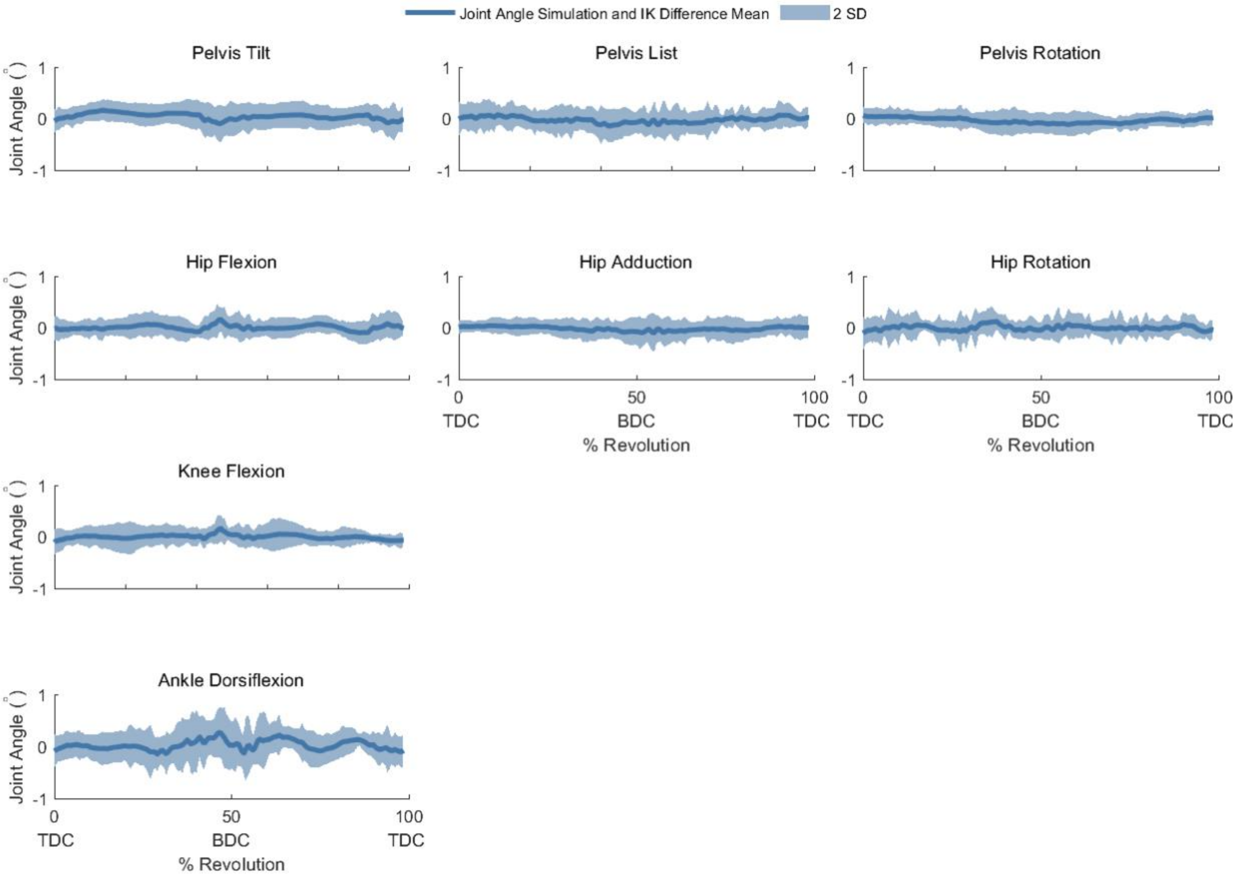


**Supplementary Figure 4.** Mean +/- 2 standard deviations (SD) of difference between joint angles in simulation and in Inverse Kinematics (IK), n=16. Over the whole revolution, the mean difference approximated the 0-line and 2 SD of the differences were less than 1 degree.

## Subject 02

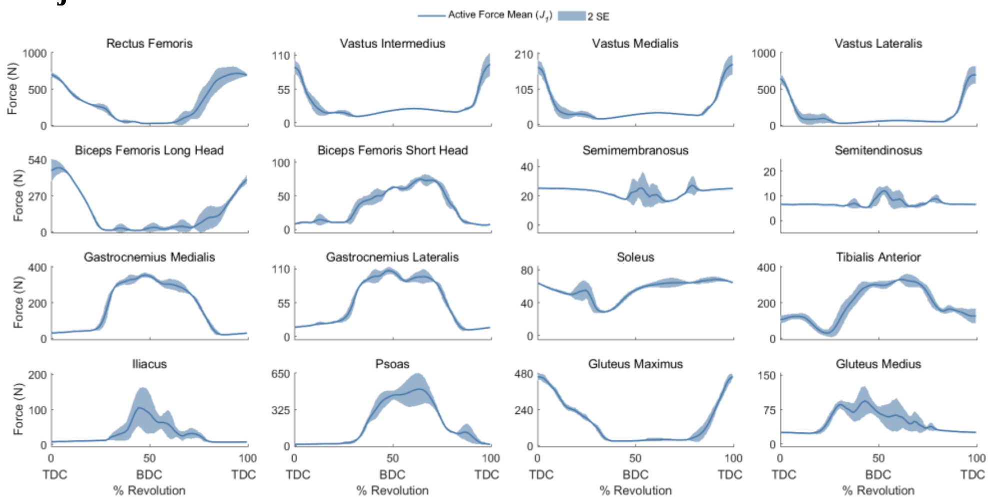


## Subject 07

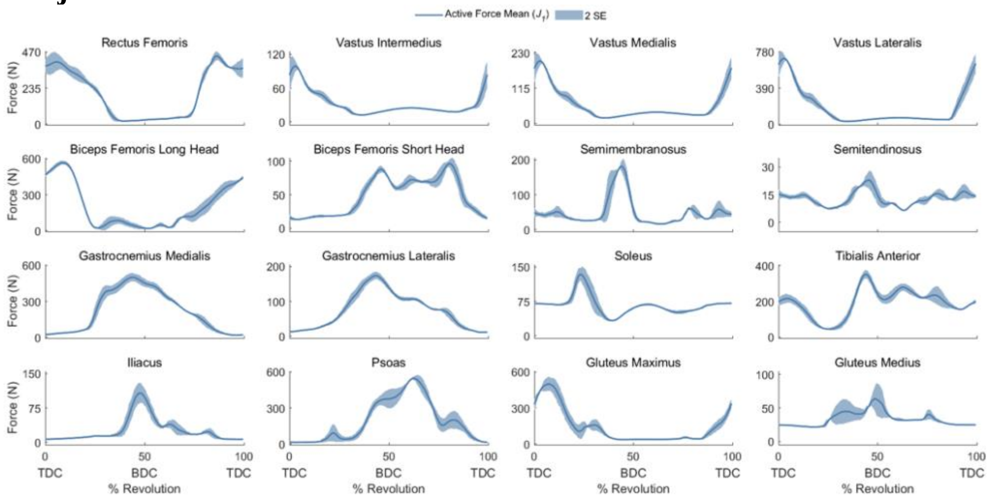


## Subject 13

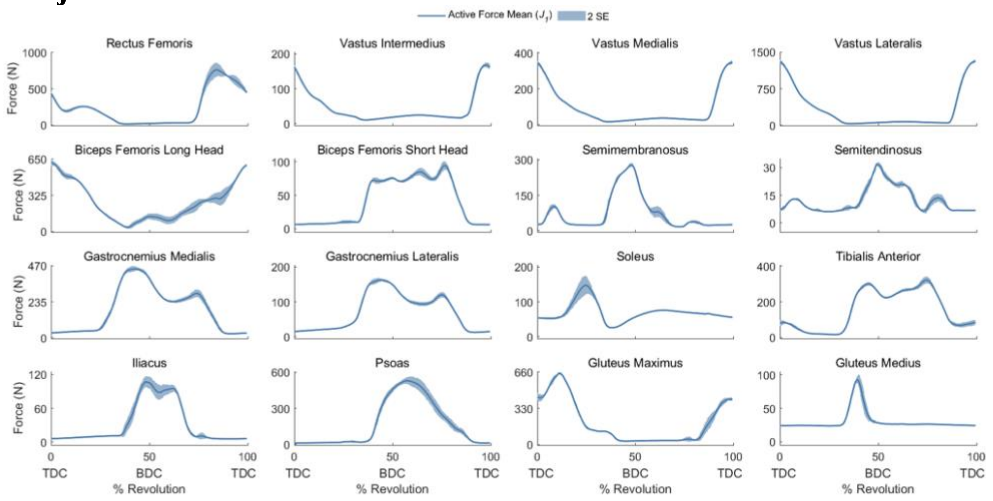

**Supplementary Figure 5.** Variations in muscle forces across three consecutive pedal revolutions from three representative participants while using the baseline objective function ($J_1$). Variations are captured as the mean +/- 2 standard errors (~95% confidence interval) for select muscles.

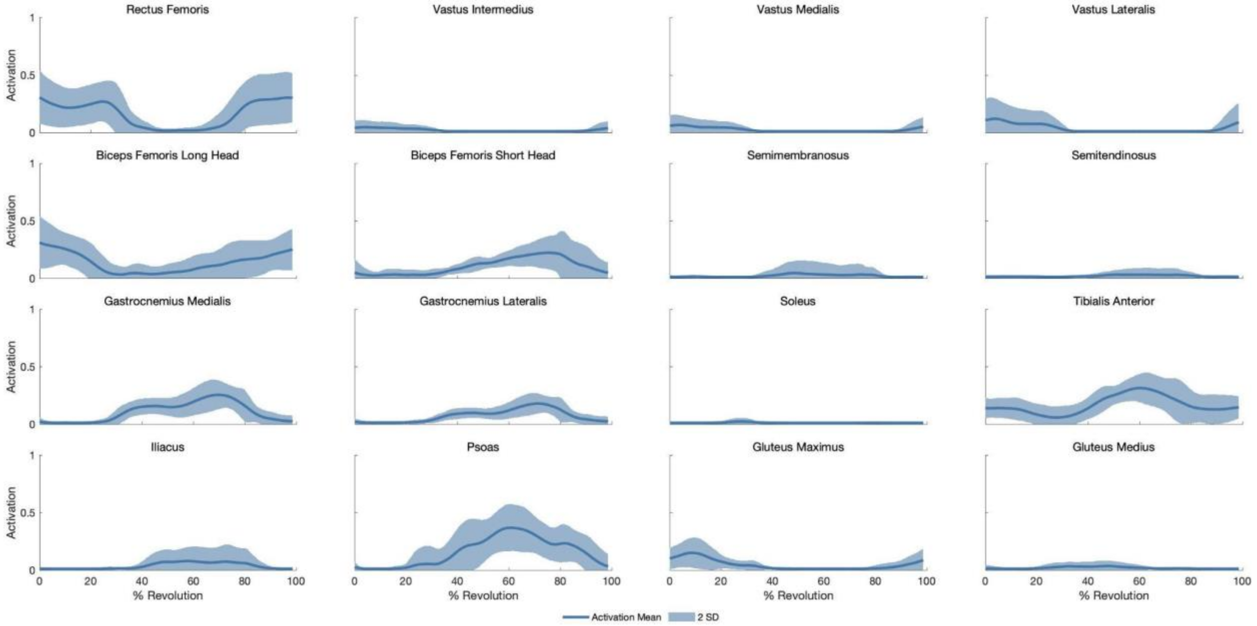


**Supplementary Figure 6.** Mean +/- 2 standard deviations for muscle activations of the right leg in baseline simulations (i.e., simulations generated using the objective function $J_1$), n=16.

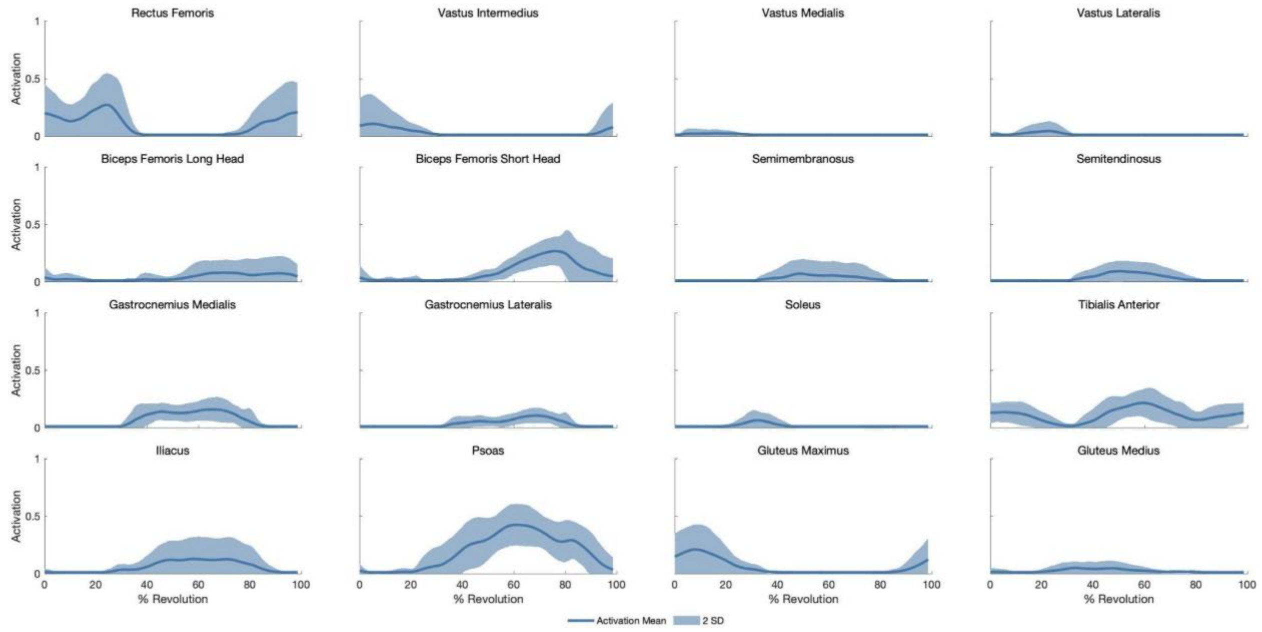


**Supplementary Figure 7.** Mean +/- 2 standard deviation for muscle activations of the right leg in simulations with an added tibiofemoral force penalty (i.e., simulations generated using the objective function $J_2$), n=16

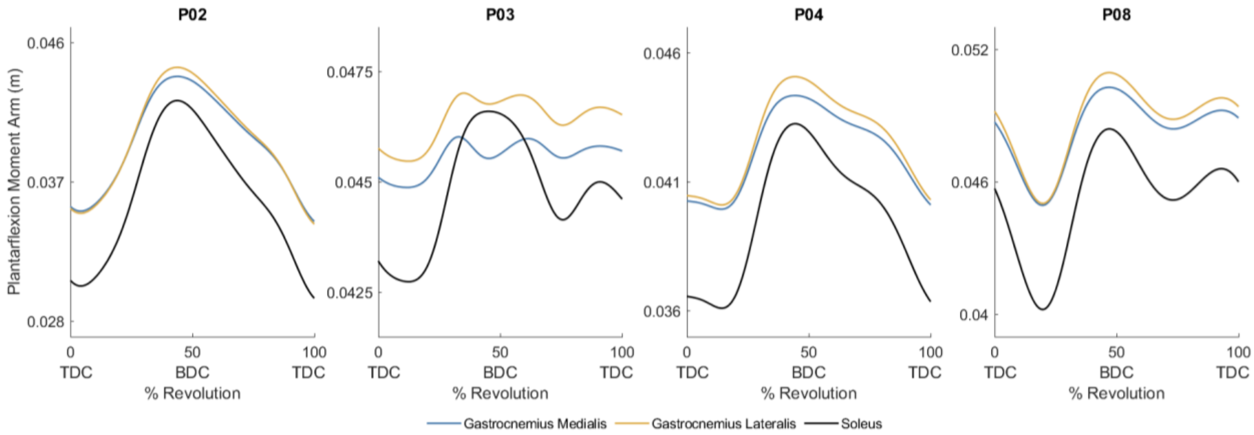


**Supplementary Figure 8.** Ankle plantar flexion moment arms over a pedal revolution for 4 representative participants. The gastrocnemii (blue and orange) consistently had a larger moment arm than the soleus (black).

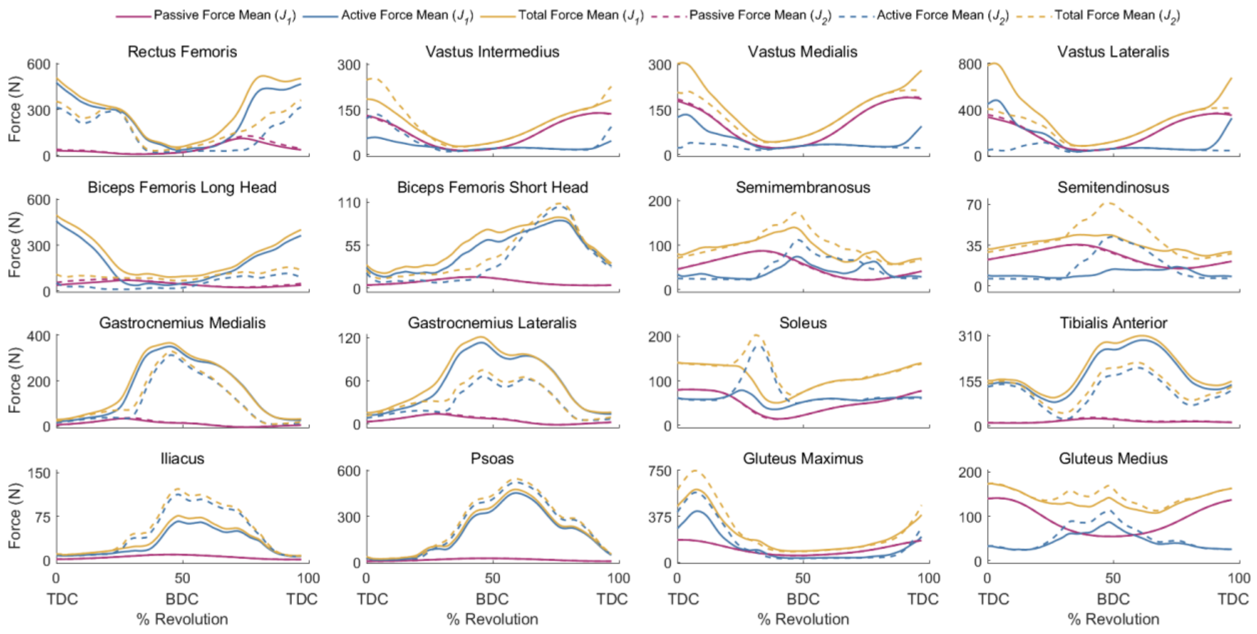


**Supplementary Figure 9.** Passive (red), active (blue), and total (i.e., active + passive; yellow) forces of the right leg in baseline simulations ($J_1$, solid lines) and in simulations with an added tibiofemoral force penalty ($J_2$, dashed lines), n=16

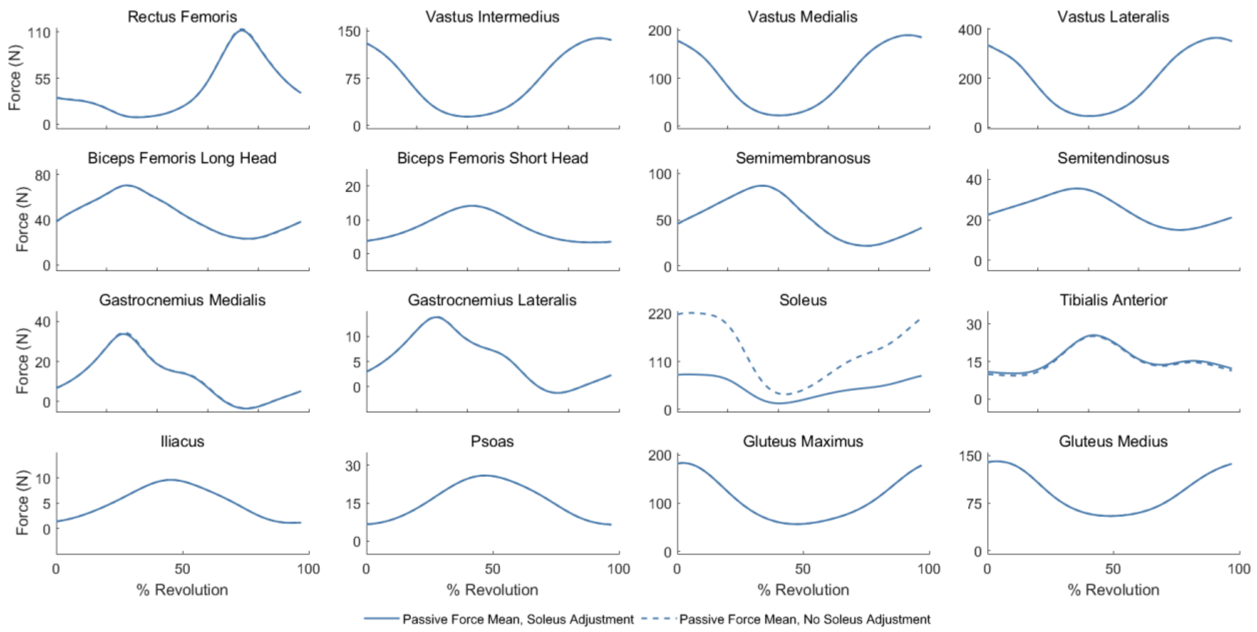


**Supplementary Figure 10.** Passive forces of the right leg with and without soleus optimal fiber length adjustment, n=16. Soleus produced high passive muscle forces during dorsiflexion angles achieved in cycling. Therefore, the optimal fiber length of the soleus was increased by one standard deviation (Rajagopal et al., 2016), as done previously to reduce passive forces in other muscles (Lai et al., 2017).

**Supplementary Discussion S1.**

Musculoskeletal model adjustments compared to related models.

To model the saddle-pelvis interaction, residual actuators were added to the pelvis with the "optimal force" property set to 100 N for translational DOFs or 100 N-m for rotational DOFs, so a control signal of 0.5, for example, would produce a force of 50 N or a moment of 50 N-m, respectively. These actuators could produce controls from -infinity to +infinity and thus were sufficient to support the rider's body weight. Increasing optimal force did not meaningfully change the simulation results. Reserve actuators were added to each of the rotational DOF with the "optimal force" property set to 0.1 N-m, except for hip rotation. The "optimal force" property for the hip rotation reserve actuator was set to 10 N-m because the model was deemed weak in this DOF and required additional actuation in hip rotation, as previously needed for squat and sit-to-stand simulations (Uhlrich et al., 2022). Future research should identify why the model is weak in hip rotation and investigate solutions that increase the capacity to generate hip rotation moments, such as improving the muscle path to increase moment arms or adjusting other muscle and tendon parameters that affect force generation.

The musculoskeletal models in this work were developed based on the model used for cycling from Lai, Arnold, and Wakeling (Lai et al., 2017), and adjustments we made are detailed here. The metatarsophalangeal joint and the subtalar joint were each replaced with a weld joint. Soleus produced high passive muscle forces during dorsiflexion angles achieved in cycling. Therefore, the optimal fiber length of the soleus was increased by one standard deviation (Rajagopal et al., 2016), as done previously to reduce passive forces in other muscles (Lai et al., 2017) (Supplementary Fig. 10). After scaling the model to each participant, muscle path via points of the iliacus and psoas muscles commonly penetrated their respective wrap surfaces, producing erroneous paths; where necessary, muscle path via points of the iliacus and psoas muscles were manually adjusted to ensure they remained outside of the wrap surface.

**References for Supplementary Content**

Lai, A. K. M., Arnold, A. S. & Wakeling, J. M. Why are Antagonist Muscles Co-activated in My Simulation? A Musculoskeletal Model for Analysing Human Locomotor Tasks. Ann. Biomed. Eng. 45, 2762–2774 (2017)

Rajagopal, A. et al. Full-Body Musculoskeletal Model for Muscle-Driven Simulation of Human Gait. IEEE Trans. Biomed. Eng. 63, 2068–2079 (2016).

Uhlrich, S. D. et al. OpenCap: 3D human movement dynamics from smartphone videos. http://biorxiv.org/lookup/doi/10.1101/2022.07.07.499061 (2022) doi:10.1101/2022.07.07.499061.

Buford WL, Ivey FM, Malone JD, Patterson RM, Pearce GL, Nguyen DK, Stewart AA. Muscle balance at the knee - moment arms for the normal knee and the ACL-minus knee. IEEE Transactions on Rehabilitation Engineering. 1997 Dec;5(4):367-79.
